# The Discussion of a Policy

**Published:** 1921-01-08

**Authors:** 


### THE DISCUSSION OF A POLICY.

We sympathise with Mr. T. Glenton-Kerr and the correspondents who agree with him in their desire to safeguard the Metropolitan Hospital Saturday Fund from any wasteful competition. But is there anything, necessarily, in Mr. Coles' suggestion for a general voluntary rate on the basis of the National Insurance Scheme which need conflict with Hospital Saturday? From figures previously published in The Hospital, we know that some 400,000 people in London are regular subscribers to the Hospital Saturday Fund. We also know, as Mr. Glenton-Kerr will probably agree upon reflection, that these 400,000 subscribers are not "the vast majority of weekly wage earners." If they were, Mr. Inman's strenuous efforts to add to their number would be unnecessary. It is, therefore, important to define more exactly what is meant by a "weekly wage earner," and to decide how far most of the latter class are financially above the National Insurance line, since for those above, in the words of Mr. Glenton-Kerr, "no web of collecting machinery" exists at present. The Insurance Act affects those with incomes below £250 a year. Is it seriously suggested that there are not more than 400,000 such persons in London? If not, it is plain that Mr. Coles has an excuse for suggesting that this class might make a voluntary levy in return for some of the institutional treatment which they need when ill. Before deciding upon such a levy, we should of course have to determine what the effect would be upon the public-spirited 400,000. It has proved possible, in certain neighbourhoods at all events, to have weekly contributions and charges toward maintenance. The question is, would it prove possible to have the levy suggested and contributions to the Saturday Fund? A decision cannot be lightly made; and since any flirting with State machinery usually produces unforeseen results, there is added need for caution. Consequently, we have preferred to advocate precisely that "web of collecting machinery" which Mr. Glenton-Kerr favours, except that, while he advocates it for "those above the National Insurance line," we have been content to advocate it for all unaffiliated to the Hospital Saturday Fund. In short, we believe that the vast majority of weekly wage earners do *not* contribute to the Hospital Saturday Fund, while agreeing with Mr. Glenton-Kerr that machinery is needed to collect contributions from those who do not subscribe to the Saturday Fund at present.

Weekly wage earners are the majority of people in London, whether or no their incomes are above or below £250 a year. The majority of patients in the voluntary hospitals are weekly wage-earners; and even after we have included those who subscribe to their local hospital, it is clear that the majority of these people are not among the regular subscribers. We therefore continue to urge the importance of collections from this class, whether above or below the Insurance Act limit, and believe that the unit of collection should be, not the income of the person, but the place wherein he works. A weekly collection, without regard to income, would do much. In view of the difference in income, a collection of a penny in the pound would do more. It is the duty of the employers in each place of business to start it. If this unit be taken many difficulties disappear, for decentralisation has many advantages. Whether the resulting sums should be paid to local hospitals or to a central fund, should be left to the contributors' choice. If a central fund were preferred by the majority, or even by a good proportion of subscribers, well, the Saturday Fund exists. Mr. Inman's friendly hand is always ready to give help or merely advise as the case may be. In justice to Mr. Coles, it should also be remembered that the levy is not his sole scheme.

Such confusion as has attended the recent correspondence on the subject in our columns, appears to have been caused by the rather loose use of the phrase "weekly wage earner." This person has been described as the proper province of the Hospital Saturday Fund; but, in the main, while subscribers to that fund consist of persons earning five pounds a week or less, weekly wage earners frequently earn much more. We invite our correspondents to think more of the class, and less of the precise sum earned per week. For if they will do so, we think that they will agree (*a*) that the class far outnumbers the Saturday Fund subscribers; and (*b*) that the direct machinery of the fund may be perhaps unfitted for the whole of it. Let the unit of collection therefore be, not this or that fund, nor this or that income, but the place of business. Let the employers in each district be approached by their local hospital to start collections, and the decision be left to them and to their staffs to provide the appropriate channel for its disbursement to the hospitals.

